# Oligopaints: highly efficient, bioinformatically designed probes for fluorescence *in situ* hybridization

**DOI:** 10.1186/1756-8935-6-S1-P5

**Published:** 2013-03-18

**Authors:** Brian J Beliveau, Eric F Joyce, Nicholas Apostolopoulosa, Feyza Yilmaza, Chamith Y Fonseka, Ruth B McCole, Yiming Chang, Jin Billy Li, Tharanga Niroshini Senaratne, Benjamin R Williams, Jean-Marie Rouillard, Chao-ting Wu

**Affiliations:** 1 Department of Genetics, Harvard Medical School, Boston, MA, 02115, USA; 2 Department of Biology, Boston University, Boston, MA, 02215, USA; 3 Department of Genetics, Washington University School of Medicine, St. Louis, MO 63110, USA; 4 Department of Genetics, Stanford University, Stanford, CA, 94305, USA; 5 Fred Hutchinson Cancer Research Center, Seattle, WA, 98109, USA; 6 MYcroarray Ann Arbor, Ml, 41805, USA; 7 Department of Chemical Engineering, University of Michigan, Ann Arbor, Ml, 48109, USA

## 

Fluorescence *in situ* hybridization (FISH) is a powerful tool to study chromosome structure, positioning, and gene expression on a cell-by-cell basis. We have developed Oligopaints [1], a PCR-based method for generating highly efficient FISH probes from complex DNA libraries. Our method can visualize genomic regions ranging in size from tens of kilobases to megabases with the same basic protocol and gives researchers precise control over the location and patterning of each probe set. We have mined the reference genomes of *C. elegans*, *D. melanogaster*, *A. thaliana*, *M. musculus*, and humans for genomically unique 32-base sequences with thermodynamically desirable hybridization properties, and have made these sequences available on the Oligopaints website [http://genetics.med.harvard.edu] along with a suite of scripts and documentation that will assist researchers with probe set design and allow our technology to be extended to any organism whose genome has been sequenced. Oligopaints robustly label chromosomes both in tissue culture cells and whole-mount tissue preparations and can be generated using standard molecular biology techniques and equipment at a price well below the cost of commercial FISH probes. The flexibility offered by our bioinformatic design platform has allowed us to perform complicated hybridizations, such as the simultaneous targeting of RNA and the genomic DNA flanking its site of transcription. Thus, we anticipate that Oligopaints will be a valuable tool for the study of nuclear architecture and the relationship between chromosome positioning and gene expression.
